# Supplementary material for: Using R Shiny to develop a dashboard using IPEDS, U.S. Census, and bureau of labor statistics data
Source: PLoS One. 2023 Jan 11;18(1):e0278573. doi: 10.1371/journal.pone.0278573 (PMC9833598; doi:10.1371/journal.pone.0278573)
Supplement: S1 Table — (DOCX) [file pone.0278573.s001.docx]

**S1 Table 1**

R package libraries used to develop the project

| Library | Function | Site |
| --- | --- | --- |
| data.table | Data table management and aggregation | https://cran.r-project.org/web/packages/data.table/data.table.pdf |
| dplyr | Data manipulation | https://www.rdocumentation.org/packages/dplyr/versions/0.7.8 |
| DT | Develop data tables in a Shiny application | https://rstudio.github.io/DT/ |
| formattable | Formats to improve readability | <https://cran.r-project.org/web/packages/formattable/formattable.pdf> |
| ggplot2 | Generate plots | https://ggplot2.tidyverse.org/ |
| ggpmisc | Extension of ggplot2 for graphic improvements | https://cran.r-project.org/web/packages/ggpmisc/index.html |
| ggpubr | Extension of ggplot2 for published graphics | https://rpkgs.datanovia.com/ggpubr/ |
| ipeds | Access the IPEDS data | https://ipeds.r-forge.r-project.org/ |
| leaflet | Generate interactive graphs | https://cran.r-project.org/web/packages/leaflet/index.html |
| leaflet.extras | An extension to leaflet for extra functions and features | https://cran.r-project.org/web/packages/leaflet.extras/index.html |
| plotly | Generates plots with tooltips, interacts with ggplot2 | https://plotly.com/r/ |
| RODBC | Query data from Access Files or similar databases | https://cran.r-project.org/web/packages/RODBC/RODBC.pdf |
| shiny | Generate interactive applications, the base of the dashboard | https://shiny.rstudio.com/ |
| shinydashboard | An extension to Shiny for dashboard development | https://rstudio.github.io/shinydashboard/ |
| shinyWidgets | An extension to Shiny for widget development | https://cran.r-project.org/web/packages/shinyWidgets/index.html |
| tidycensus | Query data from the U.S. Census Bureay | https://walker-data.com/tidycensus/ |
| tidyverse | A wrap of several data munging packages | https://www.tidyverse.org/packages/ |
