# Supplementary material for: Using R Shiny to develop a dashboard using IPEDS, U.S. Census, and bureau of labor statistics data
Source: PLoS One. 2023 Jan 11;18(1):e0278573. doi: 10.1371/journal.pone.0278573 (PMC9833598; doi:10.1371/journal.pone.0278573)
Supplement: Attachment — Submitted filename: Response_to_Reviewers2.docx [file pone.0278573.s002.docx]

April 29, 2022

PLoS One Editorial Board

1265 Battery Street

Suite 200

San Francisco, CA 94111

To the Editors and Peer-Reviewers:

This letter is regarding manuscript PONE-D-21-37932R1. First, we would like to thank the editors and reviewers for their time and focus on our paper titled, “Using RStudio to Develop a Dashboard using IPEDS, U.S. Census, and Bureau of Labor Statistics Data.” We would like to emphasize that this letter does not dispute any of the feedback we received on the paper or on the dynamic dashboard application. In fact, this letter acknowledges all suggestions and edits and addresses how each were approached in the attached revised manuscript. Further, we would like to note that the feedback was greatly appreciated as we are always looking for ways to improve this work and have found submitting to PLoS One rewarding because of the feedback we have received. Therefore, we give our sincerest thanks to the reviewers and editors for the time and thought they put into examining our work.

Second, we would like to state that we have created an RPubs page on which to share our programming and code. The submitted version of the manuscript and the updated RStudio application both contain links to this page where the reader will be able to view all of the programming code that was used to generate the dashboard. We mention this because this will not require any accession numbers or DOIs. All available data and programming language will be accessible through the application, or through the link to RPubs which is here: <https://rpubs.com/IPEDS>

In addition, we have included download buttons on the application itself where the user can download all datasets from the application. The combination of the programming code and the data download buttons allows for open-source data access.

We found all of your feedback valuable and used it to improve our paper and the dashboard itself. The following provides point-by-point overview of our response to your feedback.

**Formatting feedback from editor:**

Please ensure that your manuscript meets PLOS ONE's style requirements, including those for file naming. The PLOS ONE style templates can be found at https://journals.plos.org/plosone/s/file?id=wjVg/PLOSOne_formatting_sample_main_body.pdf and https://journals.plos.org/plosone/s/file?id=ba62/PLOSOne_formatting_sample_title_authors_affiliations.pdf

**Response:**

We reformatted the manuscript to use Vancouver Brackets style of formatting and followed the PLOS guidelines.

**Software feedback from editor:**

Please note that PLOS ONE has specific guidelines on software sharing (http://journals.plos.org/plosone/s/materials-and-software-sharing#loc-sharing-software) for manuscripts whose main purpose is the description of a new software or software package. In this case, new software must conform to the Open Source Definition (https://opensource.org/docs/osd) and be deposited in an open software archive. Please see http://journals.plos.org/plosone/s/materials-and-software-sharing#loc-depositing-software for more information on depositing your software.

**Response:**

This paper presents a web application that was programmed using R open-source software. We reviewed this policy and find it to be open source and available through a publicly accessible web address.

**Software feedback from editor:**

We note that you have stated that you will provide repository information for your data at acceptance. Should your manuscript be accepted for publication, we will hold it until you provide the relevant accession numbers or DOIs necessary to access your data. If you wish to make changes to your Data Availability statement, please describe these changes in your cover letter and we will update your Data Availability statement to reflect the information you provide.

**Response:**

We have included a link to the data in the application and a link to the programming code to get the data on an RPubs web page. Both the application and the web page are publicly accessible. We also address this in a draft of our new cover letter and in the manuscript.

**Reviewer 1 Comment 1:**

My main feedback would be to clarify the purpose and scope of the study. Their dashboard is functional, but it is not clear why would this paper need to be published at PONE? Are the authors interested in teaching researchers to develop similar applications? Are they interested in easing access to these data sources? If the latter is true, I recommend adding a functionality to download the data merged/compiled from their platform. This would be useful for the incorporation of these indicators from a multiplicity of data sources. I can definitely see master’s students taking advantage of this resource.

**Response:**

We identified with this feedback as we grappled with that question ourselves, and after much discussion, have decided to emphasize the main purpose of the paper as a demonstration of how to develop the application including data querying, munging, and programming. You will see this change of focus in the revised manuscript. We also emphasize that the programming and approach may be applicable to other datasets.

**Reviewer 1 Comment 2:**

Why are their merging approaches limited to two institutional types (public 2- and 4-year colleges)?

**Response:**

We explained how and why two categories were used and that other programmers could code more by studying the accompanying website with our code on it. We coded two categories because IPEDs generally classifies institutions with several levels and by reducing the code to two categories we are able to demonstrate recoding programming language. Again, the reader could use this to code any number of categories desired. We point this out in the paper as well.

**Reviewer 1 Comment 3:**

I am also wondering if the authors have considered relying on the college scorecard (https://collegescorecard.ed.gov/data) data for this resource has been standardized and compiles other data sources. This comment is based on the fact that the IPEDS data need to be standardized when conducting cross-sectional analyses. Although I understand that the current application relies on one academic year, feeding the application from the college scorecard may be a nice addition.

**Response:**

This is an excellent resource and many of the data elements available (e.g. ACT/SAT scores) on the scorecard are actually (and coincidentally) the source for a project in the works to develop an additional dashboard to examine those data. We considered these data when first developing this project and agree that the college scorecard is an excellent resource that does use some of the IPEDs data elements that we used in our analysis. The most recent data dictionary of the scorecard notes that many of the Treasury elements have been discontinued. We found it easier to use the *tidycensus* package to get county level elements regarding income and other demographics. In addition, we note your comment about standardization and did not find that necessary for this project as we just needed descriptive values for our outputs. However, our future project (as noted above) will need this and thus requires us to consider these data elements. We are specifically interested in the *rscorecard* package and API and that project is under way. This will lead to a later paper and project.

**Reviewer 2 Comment 1:**

I'm not sure the purpose of the paper is entirely clear: is it to describe how someone might create a dashboard like this? Or is it to describe the contents of this particular dashboard? At the moment it seems like a bit of a blend, but I found that hard to follow since the audience for the two uses would likely be quite different. If possible, I would try to narrow the focus to one or the other. If trying to describe *how* someone could build a dashboard like this, there needs to be more focus on how to develop a shiny dashboard. For example, the basic concepts like the `ui` and `server` were not well explained in the text. Similarly the `input$` `output$` system in {shiny} was not explained. Additionally, if this is the focus, less is needed on the background about the particular datasets chosen to integrate here (since presumably the next user would not be building this exact dashboard), and more attention could be paid to the general concepts of downloading, munging, and merging data together (with these datasets as a particular example, but not the main focus). If, on the other hand, the purpose of this paper is to *describe* the contents of this particular dashboard, then the focus on the code is not as necessary.

**Response:**

The first reviewer’s concern aligns with that of the second reviewer. Therefore, re-clarified the purpose of this dashboard in the revised manuscript. We do provide an overview of the datasets for context, but we emphasize that the user could apply this to other datasets.

We also went through the paper and made significant additions in the methods section to describe many of the functions and the logic of shiny. We also added the entirety of our programming code as a link.

**Reviewer 2 Comment 2:**

It is not entirely clear to me what the purpose of this application is. Is it just to allow users to explore this data? If it is for analysis (but presumably for people who are less keen on pulling all of the data themselves) it seems like it would need to have the ability to subset the large datasets for download. Currently, it seems all the user can do is calculate the correlation between two variables or compare a few variables between institutions.

**Response:**

We have decided to focus the emphasis on how to program dashboards using publicly available data sets. We retain information about the datasets used but articulate that our methods are applicable to other datasets. We also included the ability to pull the data and examine the code if the user desires to use it for their own purposes. We also demonstrate how to include a download handler in the programming of the application.

**Reviewer 2 Comment 3:**

The R code was hard to read and a bit inconsistent style-wise. I recommend using a linter to keep the code consistent (for example: <https://github.com/r-lib/lintr>)

**Response:**

We ran both the data munging file and the application through *lintr* and addressed several formatting details given the specific suggestions of Hadley Wickham. This helped us clean up our code on such details as visible binding for global variables, spacing issues (particularly around infix operators), trailing blank spaces, and trailing white spaces. We incorporated these changes to the paper, but more importantly, wrote them on the RPubs page using *knitr* on RMarkdown. We did not address 80 character spaces in our HTML code, and we did not make all variable and function names snake case, the latter mainly for functionality purposes. In addition, we utilized page-break to keep the chunks of code together so they don’t split pages. We did not use track changes when adjusting the code in the revised manuscript as we felt it was too confusing.

**Reviewer 2 Comment 4:**

I'm not sure "RShiny" is how RStudio would refer to this product (I think just Shiny for the product {shiny} for the package?).

**Response:**

We have adjusted this throughout the manuscript.

**Reviewer 2 Comment 6:**

Since the purpose of the paper is not entirely clear, I'm not sure if this review includes a review of the dashboard itself, but if so, here are a few comments:

**Response:**

We addressed all of these comments in the following list. These were very helpful in improving our application.

- On the "Institution map" page it says to "use the search box", however I do not see a search box on this page (other than the magnifying glass on the map itself?)
  - This is a great observation and our original directions could well have been confusing to users. We have therefore changed the text to say “magnifying glass” instead of “search box”.
- On the "Historic Enrollment" page, the x-axis could be cleaned up to just say 2015, 2016, 2017, 2018 (instead of `2015_Enrollment` etc.). Additionally, when more than one school is added, the numbers are completely obscured by the points on the graph. Either the points could be removed (and replaced with just the numbers), the numbers could be moved up a bit, or the user could just hover to see the numbers.
  - The dates have been addressed by recoding the value labels in the dataset. We agree that this improves the look and feel of the application.
  - The obscured numbers was an excellent observation. We eliminated this problem as suggested by eliminating them and leaving it to the tool tip and examination of the axis.
- On the "Demographics" page, the y-axis could be cleaned up (to remove the underscore and also the word "Percent" since it is redundant with the x-axis). The ordering of the bars could be improved (maybe ordered by frequency based on the top selection?) to make it easier to read for a viewer. It is also hard to compare categories between institutions - maybe position_dodge rather than a facetted chart would make sense? Otherwise, perhaps flipping the direction so the categories are aligned across institutions to make it easier to compare between them. (The same for the Graduation and Retention tab)
  - The y-axis labels have been cleaned as suggested and it is much improved.
  - We used “position = ‘dodge2’” and replaced that with the facet_grid. We also got rid of the numbers like the enrollment report and kept them in the tooltip. This feedback significantly improved the graph.
  - We tried to order by frequency by using the *reorder()* function and that worked well with one institution, but we would get an error when the maximum category was not in agreement between institutions. For example, if the highest was percent women for institution A but the highest was white for institution B, then it would generate an error.
- In the "Dynamic Scatterplot" page, it is not clear why the points are colored by location type? It also would be nice to have a direct link on this page to the Data Dictionary since that is necessary to know what the X and Y variables are. Additionally, it would be nice to output the results in a Table rather than the `lm` output directly, if possible, since the intended audience is likely not familiar with R (it seems).
  - We created a download button to get the data dictionary on this page.
  - We were unable to put the output results in a table. However, we re-coded the text and simplified the results so that the r-squared values were much easier to read and the intercepts and coefficients were cleaner without all the programming code in the way to distract.
  - However, with the adjustments with position dodge, this is improved.
- In the "Correlation Coefficients" page "variable" is forced to stay in the selection. It is trivial to force this in the dataset without relying on the user to not delete it (create a string of the names to pass to the selectizeInput that removed "variable" from the choice and similarly when picking the variables on the server side add "variable" back in). It would also be nice to have a direct link to the Data Dictionary from this page.
  - We implemented code to eliminate this from the fixed variable and forced an unfilterable string variable as row labels using this coding logic: rownames(corrmatrix) <- corrmatrix$variable
  - This was an element that bothered us, so we are glad that it was pointed out.
  - We included a button to download the data dictionary as well as a link.
  - We also included this coding logic in the manuscript and on the RPubs site.

Thank you once again for your feedback.

Sincerely,

Authors
