# Supplementary material for: Using R Shiny to develop a dashboard using IPEDS, U.S. Census, and bureau of labor statistics data
Source: PLoS One. 2023 Jan 11;18(1):e0278573. doi: 10.1371/journal.pone.0278573 (PMC9833598; doi:10.1371/journal.pone.0278573)
Supplement: Attachment — Submitted filename: Response_to_Reviewers.docx [file pone.0278573.s003.docx]

October 23, 2022

PLOS One Editorial Board

1265 Battery Street

Suite 200

San Francisco, CA 94111

To the Editors and Peer-Reviewers:

We would like to thank you all for the time and attention you have given to our project and paper. We have reviewed your recommendations and feedback carefully and feel we now have a much better paper and dashboard. You will find our responses to your feedback in this document.

**Responses to Feedback**

**Journal Requirements:**

Please review your reference list to ensure that it is complete and correct. If you have cited papers that have been retracted, please include the rationale for doing so in the manuscript text, or remove these references and replace them with relevant current references. Any changes to the reference list should be mentioned in the rebuttal letter that accompanies your revised manuscript. If you need to cite a retracted article, indicate the article’s retracted status in the References list and also include a citation and full reference for the retraction notice.

**Response:**

We have reviewed all our references and found nothing retracted. We only had to change the order. This is tracked.

**Reviewer 2**

1) In the abstract, it looks like "R Shiny" was replaced with "RStudio" -- this may have been due to my previous comment be unclear -- I think this should say "R shiny dashboard", not "RStudio dashboard"

**Response:**

We changed it to say “Using R Shiny to Develop a Dashboard . . .”

2) There are inconsistencies in how Shiny is referred to (for example RShiny, R shiny, RStudio, shiny etc) I would recommend replacing these all with "Shiny" when referring to the application and "shiny" when referring to the package.

**Response:**

We went through it and changed all of this to Shiny.

3) The detailed background on the datasets does not seem necessary, particularly the historical context. The information on what the datasets provide, however, seems relevant.

**Response:**

We removed historical content from the document.

4) I found the following explanation of a pipe confusing:
   "You’ll also notice the use of “%>%” in the code. That is a pipe that basically tells R to use the dataset that was previously stated, or to continue processing given what was done before."
   Perhaps something like:
   "The %>%, known as the "pipe" is a function from the magrittr package. It takes the object on the left hand side and "pipes" it into the first argument of the subsequent function. For example institutioninformation %>% select(UNITID) is equivalent to select(institutioninformation, UNITID). A benefit of the pipe is it can allow the code to be more readable than a series of nested functions."5)

**Response:**

We really liked how you put that and adapted your words. This was very helpful. You’ll notice we wrote:

“The “%>% is known as a pipe and is a function of the magrittr package. This function takes the object on the left hand side and “pipes” it onto the first argument of the subsequent function. For example, *institutioninformation %>%select(UNITID)* is equivalent to *select(institutioninformation, UnitID*). The pipe allows the code to be more readable than a series of nested functions.”

5) Perhaps explain what the tidyverse is, i.e. "Once the data are queried, it may be necessary to recode variables using tidyverse, a suite of R packages used to manipulate data frames".

**Response:**

We adopted your language here again, changing only one word:

“Once the data are queried, it may be necessary to recode variables using *tidyverse*, a suite of R packages used to clean and munge data frames”

6) There are several times the authors refer to assigning an object in R as "names the table" -- technically this should be something like "a table is created called x" since the process is actually creating the table and naming the object something rather than just assigning a name.

**Response:**

We went through the paper and changed the language accordingly.

7) The code on page 17 could be reduced to a series of left joins connected by the pipe (rather than creating something named "census" over and over again, ie:

census <- left_join(employ, Health, by = "GEOID") %>%
rename(GEOID = GEOID.x) %>%
left_join(income, by = "GEOID") %>%
left_join(TotalWhite, by = "GEOID") %>%
left_join(Veteran, by = "GEOID")

**Response:**

The code has been changed to this. As a side note, we started piping our newest code this way. We used to do it the longer way to detect errors better, but decided your suggestion was better.

We also fixed this with the IPEDS data joins.

ipedsdashdata <- left_join(institutioninformation, enrollmentinformationgender, by = "UNITID")%>%

left_join(enrollmentinformationrace, by = "UNITID")%>%

etc.

We also changed it in the RMarkdown and republished it.

8) Small typos:
   a. When referring to a function in text, it generally should have the open and closed brackets (or no brackets at all) (i.e obcDriverConnect( should be obcDriverConnect() or obcDriverConnect)
   b. Page 15 look says "install. Packages" should be "install.packages"
   c. Page 22 "page gives the complete programming language" should read "page gives the complete programming code"
   d. Page 25 HTLM -> HTML

**Response:**

a. We went through all the brackets and closed any open ones.
b. We changed it to “install.packages”.
c. We changed “language” to “code”.
d. We changed “HTLM” to “HTML”

**Reviewer 3**

1) What process did the authors use to select these variables and not others to appear in the dashboards? There are many data points left out.

**Response:**

The purpose of this paper was to demonstrate the development of this dashboard. The variables chosen for inclusion in the dashboard are meant to inform higher education administrators, institutional researchers, and postsecondary policymakers. However, this paper is intended to inform the reader how a similar dashboard using large, publicly available datasets might be created. We encourage the readers of this paper to study all the data sources and dictionaries of any dataset they are interested in using. To address this, we added this language to the paper:

” These datasets contain thousands of variables. IPEDS has up to 250 variables [4], the USCB over 18,000 [3] and BLS has several datasets containing tens of thousands of variables (would could find no final number) [5]. For this demonstration, we choose variables related to demographics, educational outcomes and labor outcomes. However, other programmers may choose to use any number of variables from these datasets depending on the goals of their project. No matter what variables are chosen, it is imperative that the programmer studies the variable sources, code books, and logic. This is the case whether they choose these demonstrated datasets, or other datasets. It is our goal to use the variables in this paper to demonstrate basic skills that could be applied to any number of variables from any number of data sources”

2) A main claim in the paper is that there isn’t a dashboard cited in the literature that combines US federal census, labor and education. Are there any studies that combine this data themselves without a dashboard? This would strengthen the argument that this dashboard was needed and provide a basis to evaluate the effectiveness of this dashboard in future years.

**Response:**

On our first submission, we had some literature review on the few sources that we found that combine these types of data. We have re-introduced a short paragraph about those as we agree it adds to the context or need. You will note a new section titled “These Data Sets Combined”.

3) Some discussion is needed about the logistics of hosting and maintaining this dashboard. The authors seem to be using a free account on shinyapps.io. What are the limitations as the dashboard is used more frequently for this approach? Did the authors consider other deployment paths? (i.e. was cost the only factor, or were other deployment options evaluated).

**Response:**

We have added a section on deployment and hosting of the dashboard called “Deployment of the Dashboard”. We note that the free version of Shiny comes with a data limit and that there are purchasable subscriptions if more data needs to be stored. We also note that this demonstration allows for the creation and deployment of a Shiny application. R does offer other applications where this code may be useful, but the focus of this project was to use Shiny since it is free and open-source.

4) What plans are there to maintain the data set and what is the workload? i.e. what effort is needed to add 2020 data and beyond? How long will that take given the initial code is in place?

**Response:**

We have added a section at the end called “Maintenance and Updates” where we discuss the need to update the data each year. We provide a schedule of when Census, BLS, and IPEDS data are updated and suggest updating all of the simultaneous with IPEDS data. We note that updating the data requires an adjustment of term codes in the programming language and the obtainment of the most recent data sets from each entity. We also note that with practice, updating the data need not take more than a few hours a year.

5) The response to a prior reviewer comment about 2-year or 4-year institutions I think misses the mark. This is a somewhat misleading variable in IPEDS that actually can lead to wrong conclusions if a researcher is unaware what it represents. IPEDs codes institutions as 4-year institutions if they have any bachelor’s degree programs. 2-year institutions (in the IPEDS data set) are those institutions with only associates degrees. Most times this is not actually what a researcher actually wants and excludes nearly all community & technical colleges in some states that award primarily associates degrees and less than two year certificates, but have a few bachelor’s degree programs.

**Response:**

We agree with you and have added language that encourages the user, if wanting to use this variable, to study the IPEDS data dictionary on the ACCESS file. Under the “valuesetsYY” tab, you can look up the CYYSZSET variable in the HDYYYY table and note the different categories as they are coded by IPEDS. These include a range of categories from “Two-year, very small” for a coded value of 1, to a “Not applicable” category for a value of 20. We thus agree that this is a misleading variable if the researcher wants to use IPEDS data for other purposes and have added language in our paper to emphasize that the researcher or coder should consider the limitations of the variable names in IPEDS or in any other source, but for purposes of demonstrating how to reprogram variables in R, we have used this variable.

You will also note that we included our coding logic for that variable in the paper, which is followed by this statement:

“It is important to consider the objectives of a dashboard when conducting research with IPEDS or other data. For example, this particular dataset may not include every category of higher education institutions. For example, some institutions that are not public, only award less than two year certificates, or other types may not be classified in the desired way by IPEDS or other data sources. Therefore, it is imperative of the researcher to choose datasets that meet their dashboard’s objectives.”

Thank you once again for your feedback.

Sincerely,

Authors
